# Recovery estimates and prognostic factors for oculomotor nerve palsy

**DOI:** 10.1055/s-0046-1827066

**Published:** 2026-08-13

**Authors:** Daniel Lucio Willing, Alice Giotta Lucifero, Marcos Devanir Silva da Costa, Juan Carlos Ahumada-Vizcaíno, Raphael Wuo-Silva, Feres Chaddad-Neto

**Affiliations:** 1 Universidade Federal de São Paulo, Departamento de Neurologia e Neurocirurgia, São Paulo SP, Brazil.; 2 Harvard Medical School, Brigham and Women's Hospital, Boston MA, United States.; 3 Università of Pavia, Dipartimento di Scienze del Cervello e del Comportamento, Pavia, Italy.; 4 Hospital Beneficência Portuguesa de São Paulo, Departamento de Neurocirurgia, São Paulo SP, Brazil.

**Keywords:** Oculomotor Nerve Diseases, Oculomotor Nerve, Treatment Outcome, Prognosis

## Abstract

**Background:**

Third cranial nerve (III CN) palsy is an extremely disabling condition with several etiologies and clinical onsets. Identifying the underlying causes and individual risk factors is critical to determine prognosis and guide treatment.

**Objective:**

To analyze factors associated with III CN palsy, estimate recovery rates across different etiologies, and identify independent predictors of recovery, considering patient characteristics and comorbidities.

**Methods:**

A retrospective study was performed, including demographics and neurological data of a consecutive series of patients affected by III CN palsy from 2010 onwards. All patients underwent neurological examination, routine neuroimaging, and follow-up. Kaplan-Meier curve and the log-rank method were used to estimate recovery rates by etiology. Univariate and multivariate logistic regressions assessed patient-specific factors as independent predictors of recovery. Statistical significance was fixed as *p* -value < 0.05.

**Results:**

Overall, 50 patients were included. The prevailing causative factors were microangiopathy in 36% and miscellaneous in 36% of cases. Hypertension (50%), diabetes mellitus (34%), and cardiovascular diseases (14%) were the predominant comorbidities. Total recovery of the oculomotor function was found in 46% of patients. Kaplan-Meier analysis reported microangiopathy and syndromes as etiological factors significantly related to early recovery time. Age and pupillary involvement emerged as significant independent prognostic factors.

**Conclusion:**

The etiology significantly influences the recovery estimates of the oculomotor function. At the same time, patients' age, pupillary involvement, and especially hypertension as a comorbidity were the main predictive factors of good outcomes. More extensive studies are required to confirm these findings and include then in clinical practice.

## INTRODUCTION

The third cranial nerve (III CN), also known as the oculomotor nerve, has both somatic and autonomic functions. It innervates four extraocular muscles: elevator palpebrae superioris, ciliary muscles, and sphincter pupillae. The III CN controls the ipsilateral eye movements, pupillary constriction, and upper eyelid elevation, and governs visual tracking and gaze fixation. 1 2 3 4

The true incidence of idiopathic or acquired III CN palsy largely remains uncertain. It is presumed to account for approximately 4 cases per 100,000 cases per year, mainly in patients older than 60 years. 5 While rare, neuropathies of the oculomotor nerve can lead to severe disability, which greatly affects patients' quality of life.

Literature shows that the most common causes are idiopathic, metabolic, and vascular conditions. They also include aneurysms, autoimmune diseases, trauma, as well as both malignant and benign tumors. 3 6 7 8 9 10 11 Clinical presentations include permanent or transient paralysis, ptosis, diplopia, ophthalmoplegia, ocular deviation, and pupillary dysfunction. 7 12 13 14

The prognosis is generally favorable, and spontaneous regression of symptoms commonly occurs within a few months. However, the considerable variability of causative factors and clinical presentations makes recovery timing unpredictable. 12 15

Reliable prognostic appraisals and recovery estimates based on etiology and comorbidities are strictly needed to orient the management and plan the treatment.

In this context, the primary aim of the present study was to perform a descriptive analysis of factors associated with III CN palsy and estimate the recovery rates over time for patients stratified by different etiology categories. A secondary aim was to identify independent predictors of recovery, considering etiologies, patient characteristics, and comorbidities.

Our purpose is to build a patient-based protocol to easily predict chances, retrieval, and possible sequelae, as well as to plan effective therapeutic management to improve symptoms and patient outcomes.

## METHODS

Overall data about demographic, clinical, and neurological outcomes of a consecutive series of patients diagnosed with oculomotor nerve palsy were retrospectively reviewed. Data on age, sex, neurological examination, comorbidities, and follow-up were obtained from patients' medical electronic records at Hospital São Paulo (Universidade Federal de São Paulo). The University's Ethics and Research Committee approved this study (no. 32646920.2.0000.5505). Given its retrospective nature, informed consent was waived.

Patients evaluated between 2010 and 2020 were included. The exclusion criteria were those under 18-years-old, incomplete chart data, and absence of outpatient follow-up.

### Diagnostic work-up, outcome evaluation, and follow-up

The time of diagnosis was set as the first neurological exam for III CN palsy. The oculomotor function was investigated using the degrees of freedom of the extraocular muscle movements, namely the depression, adduction, extortion, elevation, eyelid elevation, and pupillary reflex. Pupillary involvement and the presence of ptosis were also assessed.

All patients underwent investigations, including the lumbar puncture, magnetic resonance imaging (MRI), computed tomography (CT), and angiography.

Causative etiologies of III CN palsy were stratified into five categories: microangiopathy, miscellaneous, trauma, tumor, and aneurysm. Microangiopathy is the intended pathological alteration of the small vessel structure predisposing to ischemia. Furthermore, miscellaneous includes idiopathic causes and syndromes referred to as cavernous sinus syndrome, Tolosa-Hunt syndrome, oculomotor apraxia, or pachymeningitis. The comorbidities selected and examined were as follows: hypertension, diabetes mellitus types I and II (DM I/II), cardiovascular diseases, tumors, dyslipidemia, hypothyroidism, and benign prostatic hyperplasia (BPH), and the diagnosis was considered at the discretion of the patient self-report.

Before discharge, all the patients underwent a neurological assessment. Neurological outcomes were reported at the 1-month, 6-month, 1-year, and 2-year follow-ups. Results were listed as no improvement, total recovery, or partial recovery. Complete recovery was defined as the full restoration III CN function, including normal eyelid elevation, extraocular movements, and pupillary reactivity (when initially impaired). Partial recovery was defined as an incomplete restoration of function, with residual deficits in one or more components, such as mild extraocular muscle weakness, persistent ptosis, or abnormal pupillary response.

### Statistical analysis

The R software (R Foundation for Statistical Computing) and Prism 9 (GraphPad Software, Inc.) software were used for the statistical analysis. Continuous variables with normal distributions were expressed as mean ± standard deviation (SD), and categorical variables as frequencies and percentages.

For the first study goal, the Kaplan-Meier method was employed, estimating the recovery rates over time for patients stratified by different etiology categories. Recovery time was calculated from the date of the first hospital visit regarding the III CN palsy to the last follow-up or recovery. Kaplan-Meier survival curves were plotted, using different colors and patterns to distinguish etiological groups. The log-rank test was applied to compare differences in recovery distributions between groups, with a significance level set at *p* < 0.05.

As a second step of the analysis, univariate logistic regression analyses were conducted to identify predictors of recovery influencing the likelihood of complete recovery, including age, hypertension, pupillary involvement, ptosis, and comorbidities such as DM and cardiovascular diseases. Variables with a *p* -value < 0.05 in the univariate analysis were entered into a multivariate logistic regression model to determine independent predictors of total recovery. Odds ratios (ORs) with corresponding 95% confidence intervals (CIs) were calculated, and a *p* -value < 0.05 was considered statistically significant for all analyses.

## RESULTS

### Demographic, clinical, and neurological data

**Figure 1:**
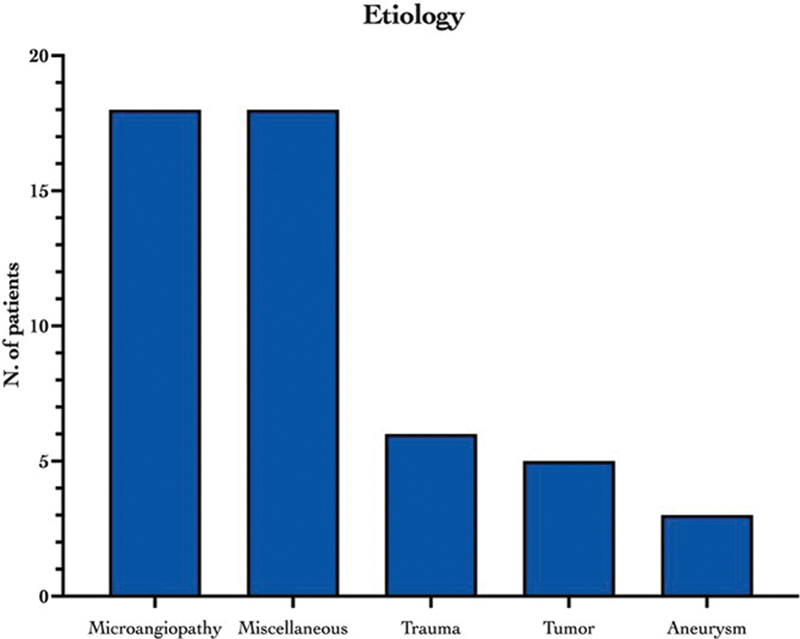
Bar graph showing the incidence of III CN palsy causative etiologies.

In total, 3,258 medical records were examined, and after the implementation of the inclusion criteria, 50 patients were retrieved in the present study. The mean age was 48.4 ± 22 years, and 21 (42%) presented right-sided III CN palsy. The leading etiologies were microangiopathy (n = 18, 36%), miscellaneous (n = 18, 36%), trauma (n = 6, 12%), tumor (n = 5, 10%), or aneurysm (n = 3, 6%), respectively ( Figure 1 ).

**Figure 2:**
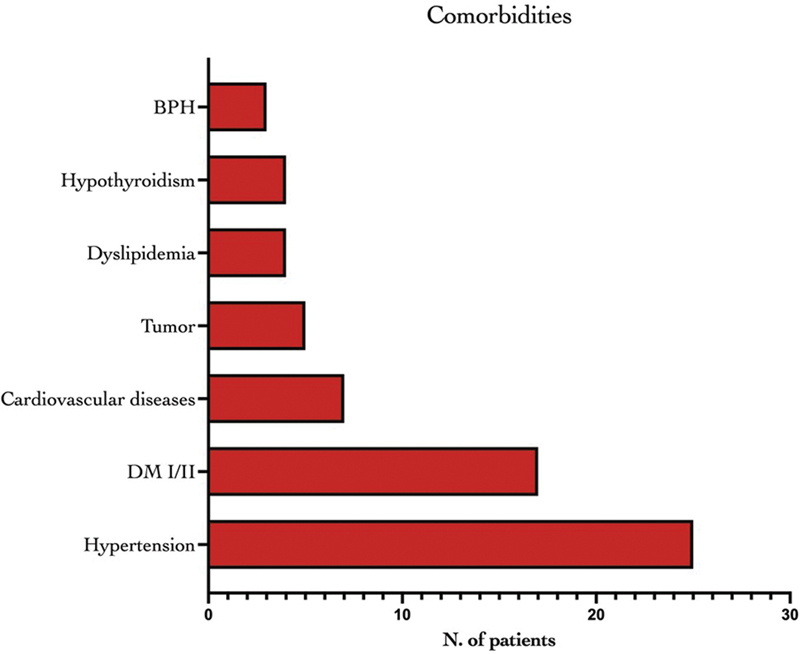
Abbreviations: BPH, benign prostatic hyperplasia; DM, diabetes mellitus; N, number. Bar graph describing the incidence of comorbidities affecting the patients' cohort.

The neurological examinations revealed ptosis in 31 (62%) patients and pupillary involvement in 15 (30%) cases. Regarding comorbidities, 25 (50%) patients were affected by hypertension, 17 (34%) presented with DM I/II, 7 (14%) cardiovascular diseases, 5 (10%) had tumors, 4 (8%) dyslipidemia, 4 (8%) hypothyroidism, and 3 (6%) patients were diagnosed with BPH ( Figure 2 ).

**Figure 3:**
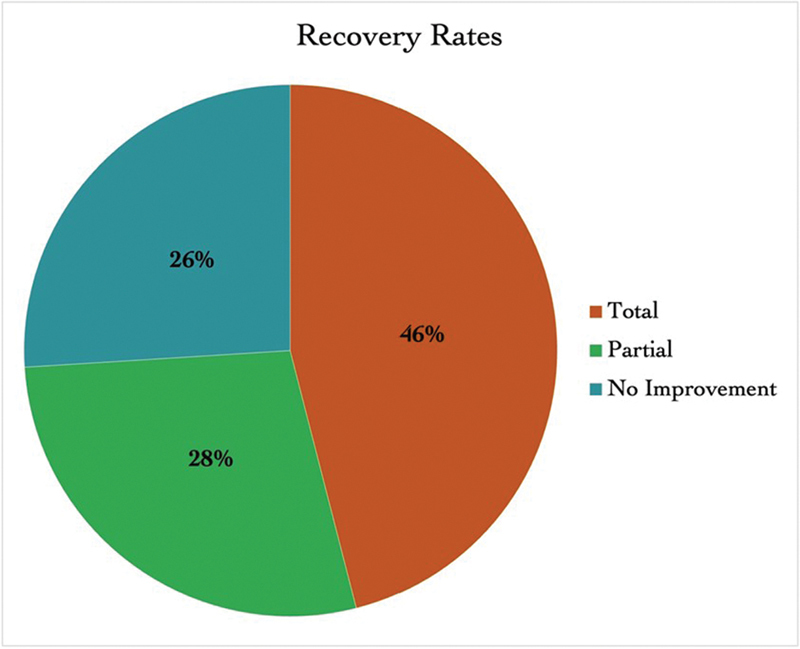
Pie graph reporting the percentage of recovery rates.

Among all patients, no improvement in III CN deficit was reported in 13 (26%) of cases, while the recovery rates were total in 23 (46%) and partial in 14 (28%) of patients ( Figure 3 ).

The average recovery time was faster for patients in the miscellaneous group or affected by microangiopathy, amounting to 1.6 and 2.8 months, respectively. The mean follow-up was 11 months for the present case series ( Table 1 ).

**Table 1:** Patients' demographic, clinical, and neurological data

Variable	Data
Timeframe	Jan. 2010–2022
Total, n	50
Average age (yrs ± SD)	48.4 ± 22
Side, n (%)	
Right	21 (42)
Left	16 (32)
Bilateral	8 (16)
NA	5 (10)
Pupillary involvement, n (%)	15 (30)
Ptosis, n (%)	31 (62)
Etiology, n (%)	
Microangiopathy	18 (36)
Miscellaneous	18 (36)
Trauma	6 (12)
Tumor	5 (10)
Aneurysm	3 (6)
Comorbidities, n (%)	
Hypertension	25 (50)
DM I/II	17 (34)
Cardiovascular diseases	7 (14)
Tumoral	5 (10)
Dyslipidemia	4 (8)
Hypothyroidism	4 (8)
BPH	3 (6)
Recovery rates	
Total	23 (46)
Partial	14 (28)
No improvement	13 (26)
Recovery times (average months)	
Microangiopathy	2.8
Miscellaneous	1.6
Trauma	36
Tumor	4.5
Aneurysm	10
Average FU (months)	11

Abbreviations: BPH, benign prostatic hyperplasia; DM, diabetes mellitus; FU, follow-up; NA, not available; N, number; SD, standard deviation; Yrs, years.

### Recovery estimation and logistic regression analyses

Kaplan-Meier analysis was conducted based on different covariates to estimate differences in recovery rates between categories of etiologies.

**Figure 4:**
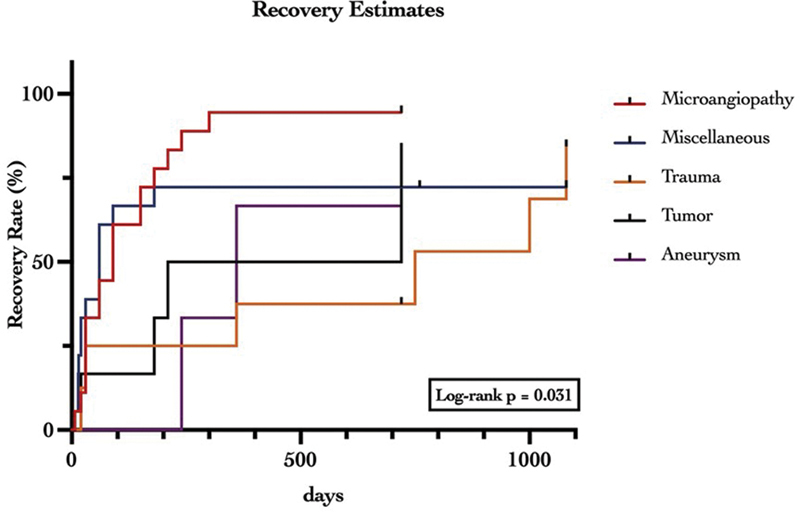
Kaplan-Meier curve of recovery estimates over different etiologies.

The curve showed a significant difference in the recovery time. As shown in Table 1 , patients with idiopathic III CN palsy or affected by microangiopathy and miscellaneous conditions recovered faster than those with tumors, aneurysms, or posttraumatic oculomotor nerve impairment. The log-rank test confirmed a significant difference in the recovery rates ( *p* = 0.031), as shown in Figure 4 .

**Figure 5:**
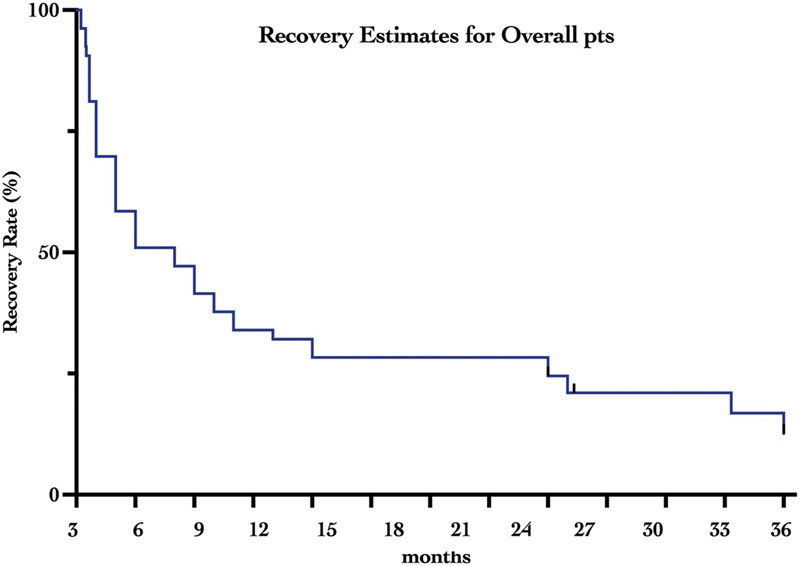
Kaplan-Meier curve of overall recovery estimates.

Furthermore, Kaplan-Meier was included showing the time of recovery for the overall sample ( Figure 5 ).

Univariate logistic regression analyses identified several clinical and demographic factors associated with recovery. Older age was significantly related to recovery outcomes (OR: 1.04; 95% CI: 1.01–1.08; *p* = 0.002), indicating that each additional year of age decreased the likelihood of complete recovery. Hypertension was also associated with a less favorable outcome (OR: 4.88; 95% CI: 1.2–24.6; *p* = 0.021). Pupillary involvement (OR: 0.06; 95% CI: 0.01–0.2; *p* < 0.0001) emerged as a strong predictor of recovery. Variables such as lesion side, DM I/II, cardiovascular diseases, tumors, dyslipidemia, hypothyroidism, and ptosis did not reach statistical significance in the univariate analyses.

In the multivariate analysis, after adjustment for potential confounders, both age (OR: 1.05; 95% CI: 1.01–1.10; *p* = 0.029) and pupillary involvement (OR: 0.02; 95% CI: 0.001–0.23; *p* = 0.005) remained independent predictors of outcome, with older age and pupillary involvement being associated with a lower probability of complete recovery ( Table 2 ).

**Table 2:** Logistic regression analyses of prognostic factors

	OR	95% CI	*p* -value
**Univariate analysis**			
Age	1.04	1.01–1.08	0.002*
Side	0.09	0.4–2	0.718
Hypertension	4.88	1.2–24.6	0.021*
DM I/II	1.37	0.3–5.8	0.648
Cardiovascular diseases	2.32	0.3–46.2	0.568
Tumors	0.48	0.07–4.0	0.459
Dyslipidemia	0.41	1.8–2.6	0.226
Hypothyroidism	1.05	0.1–22.5	0.961
BPH	0.41	1.7–2.1	0.981
Pupillary involvement	0.06	0.01–0.2	< 0.0001*
Ptosis	0.55	0.10–2.19	0.413
**Multivariate analysis**			
Age	1.05	1.01–1.10	0.029*
Pupillary involvement	0.02	0.001–0.23	0.005*

Abbreviations: BPH, benign prostatic hyperplasia; CI, confidence interval; DM I/II, diabetes mellitus; OR, odds ratio.

Notes: *In univariate analysis, age, hypertension, and pupillary involvement demonstrated statistical significance. In multivariate analysis, both age and pupillary involvement maintained statistical significance ( *p* < 0.05).

## DISCUSSION

Oculomotor nerve neuropathies are burdened by numerous underlying causative pathologies and distinctive clinical presentations related to specific anatomical features. The III CN originates from two midbrain nuclei: the oculomotor nucleus and the accessory parasympathetic/Edinger-Westphal nucleus. Its roots emerge from the oculomotor sulcus toward the interpeduncular fossa. The nerve runs parallel to the posterior communicating artery, between the posterior and superior cerebellar arteries, crosses the cavernous sinus, and penetrates the orbit through the superior orbital fissure. 2 16 17

The marked variability in clinical presentations is also attributable to the spatial layout of the fibers within the nerve. The parasympathetic fibers build up the outer layer, while the inner somatic fibers are bundled deep inside the nerve. 18 19 20 Based on the depth and anatomical location of the damage, the symptomatologic onset consists of ipsilateral diplopia, mydriasis, and ptosis. Generally, III CN deficits are incomplete and transient but can still result in significant functional disability for the patient. 20

Building on these assumptions, we retained that it is of primary importance to implement a specific early diagnosis protocol, tailored to patient characteristics, which is competent of predicting prognosis and guiding treatment planning.

In the present study, 50 patients diagnosed with oculomotor nerve palsy were analyzed through medical records. The prevailing causative factors were microangiopathy in 36% of patients and miscellaneous in 36% of cases, whereas trauma, tumors, and aneurysms were less common.

Similar to our results, Fang et al. 5 reported the most common etiological factors as microvascular (42%), followed by trauma (12%), tumors (11%), postsurgical (10%), and aneurysms (6%), in a series of 145 cases of III CN palsy. 5 In 2020, in a nationwide Korean cohort study, Jung et al. 21 examined 387 patients newly diagnosed with III CN palsy in 10 years period and identified vascular diseases as the leading cause (52.7%), followed by idiopathic causes (25.8%), neoplasms (7.8%), aneurysms (5.4%), and trauma (5.2%). 21

Regarding comorbidities, 50% of our patients had hypertension, 34% by DM I/II, and 14% by cardiovascular diseases. Indeed, literature reports diabetic peripheral neuropathy, along with cardiovascular risk factors, as the main comorbidities. 22 23 In 2010, Keane analyzed 1,400 inpatients with oculomotor palsy, of whom 53% had DM, autonomic neuropathy, and microvascular ischemia. 23

Overall, recovery outcomes in our cohort were favorable. Retrieval rates were total in 23 (46%) and partial in 14 (28%) patients in an 11-month follow-up. Kaplan-Meier analysis revealed significant differences in recovery time based on causative factors ( *p* = 0.031).

Patients with microangiopathic etiology exhibited the most rapid and highest rates of recovery, with a steep early increase indicating that the majority of recoveries occurred within the initial months of follow-up. Miscellaneous causes showed a similarly early but more gradual recovery pattern, reaching a plateau thereafter. In contrast, aneurysm-related cases demonstrated a delayed recovery profile, with minimal early improvement followed by a progressive increase over time, ultimately approaching comparable recovery rates at longer follow-up. Tumor-related deficits were characterized by a slower and more stepwise recovery, with late improvements observed, likely reflecting the impact of treatment and progressive neural adaptation. Traumatic etiologies showed the lowest overall recovery rates, with a relatively flat curve during early follow-up and only modest improvement over time. Overall, these findings indicate that both the probability and timing of recovery are strongly influenced by the underlying etiology, with distinct temporal patterns observed across groups.

Our results are consistent with the literature. In a study conducted in 2019, Chen et al. 22 analyzed 121 patients affected by oculomotor nerve palsy and reported total and partial recovery rates of 53.7 and 38.0%, respectively, at 6-month follow-up. Patients with diabetic microangiopathy showed the best outcome, with a 71.9% full recovery rate, significantly higher than that of patients with other etiologies. In 2024, a retrospective study investigating factors related to long-term recovery in CN palsy found that impairments in the third nerve were associated with shorter symptom duration and recovery periods. The most favorable recovery outcomes among causative factors were observed in cases of inflammation and ischemic events. 24

Furthermore, in our univariate logistic regression analyses, age ( *p* = 0.002) and hypertension ( *p* = 0.021) were identified as predictors of poorer outcomes, whereas pupillary involvement ( *p* < 0.0001) was associated with a lower likelihood of complete recovery. In the multivariate analysis, only age ( *p* = 0.029) and pupillary involvement ( *p* = 0.005) remained independent prognostic factors.

Proof that the elderly age may be a prognostic and risk factor in case of III CN nerve damage is closely correlated with the rate of aging. This influences chronic inflammatory diseases and impacts organs and tissues differently, reducing the chance of recovery. 6 25 In the Korean study, the incidence of III CN palsy increased after 60-years-old, peaking at 75 and 79 years. 21 The mean patients' age was 48.4 ± 22 years, confirming old age as a more frequent and prognostic factor for slower recovery.

Our clinical examinations reported ptosis and pupillary involvement in 62 and 30% of cases, respectively.

Contrary to expectations, pupillary involvement in our study was unexpectedly associated with a higher likelihood of an unfavorable outcome, which we found unexpected given that pupillary dysfunction is traditionally interpreted as a marker of more severe acute neural impairment, often related to compressive or ischemic injury.

As reported by Fang et al., compressive or vascular lesions frequently impair the pupillary function (64% of cases in their cohort). 5 This is due to compressive stress on superficial parasympathetic fibers, resulting in incomplete or complete pupillary motor dysfunction. 26

Therefore, this study investigated the effect of different etiologies and the impact of patients' features, demographics, and comorbidities on the prognosis and recovery outcomes of III CN palsy. We found that age and pupillary involvement, as well as the underlying etiologies, may influence recovery in III CN palsy.

### Study limitations

This observational study has some limitations, primarily related to the relatively small sample size. The intrinsic retrospective nature of the study increases the risk of potential selection or information biases. This study is limited by the assessment of “time to recovery.” Clinical follow-up was performed at non-uniform and sometimes prolonged intervals; therefore, the exact timing of recovery could not be precisely determined and likely occurred at an unknown point between two visits. This introduces a degree of interval censoring and may reduce the accuracy of point estimates for recovery time.

Furthermore, in clinical practice, a precise scale for assessing and estimating CN damage has yet to be reported. This last aspect may affect the evaluation of outcomes. Additional multicenter studies are needed to validate our results.

In conclusion, Oculomotor nerve palsy is a disabling condition characterized by diverse clinical presentations and different causative factors. Microvascular pathologies and syndromes proved to be the etiological factors that most positively impact recovery time. The patient's age, along with hypertension and pupillary involvement, are significant independent predictors of total or partial recovery.

Although further validations with larger samples are required, future perspectives include implementing a patient-based protocol in clinical practice to provide an early diagnosis, proper therapeutic management, and an appropriate prognosis.
